# Auto-Phase-Locked Time-Resolved Luminescence Detection: Principles, Applications, and Prospects

**DOI:** 10.3389/fchem.2020.00562

**Published:** 2020-06-30

**Authors:** Qisheng Deng, Zece Zhu, Xuewen Shu

**Affiliations:** Wuhan National Laboratory for Optoelectronics & School of Optical and Electronic Information, Huazhong University of Science and Technology, Wuhan, China

**Keywords:** time-resolved, luminescence, auto-phase-locked, chopper, imaging

## Abstract

Time-resolved luminescence measurement is a useful technique which can eliminate the background signals from scattering and short-lived autofluorescence. However, the relative instruments always require pulsed excitation sources and high-speed detectors. Moreover, the excitation and detecting shutter should be precisely synchronized by electronic phase matching circuitry, leading to expensiveness and high-complexity. To make time-resolved luminescence instruments simple and cheap, the automatic synchronization method was developed by using a mechanical chopper acted as both of the pulse generator and detection shutter. Therefore, the excitation and detection can be synchronized and locked automatically as the optical paths fixed. In this paper, we first introduced the time-resolved luminescence measurements and review the progress and current state of this field. Then, we discussed low-cost time-resolved techniques, especially chopper-based time-resolved luminescence detections. After that, we focused on auto-phase-locked method and some of its meaningful applications, such as time-gated luminescence imaging, spectrometer, and luminescence lifetime detection. Finally, we concluded with a brief outlook for auto-phase-locked time-resolved luminescence detection systems.

## Introduction

Time-resolved techniques have been widely applied to the study of ultrafast photophysical processes (Wirth, 1990; Walker et al., 2013), the research of temporal behavior of chemical systems (Piatkowski et al., 2014), biological detection and imaging (Connally and Piper, 2008; Berezin and Achilefu, 2010; Bünzli, 2010; Cicchi and Pavone, 2011; Strat et al., 2011; Becker, 2012; Yang et al., 2013; Grichine et al., 2014; Lemmetyinen et al., 2014; Lu et al., 2014; Bui et al., 2017; Luo et al., 2017; Zhang et al., 2018; Zhu et al., 2018b; Liu et al., 2019). Since these techniques can detect the luminescence change in time domain, they play a more and more important role as many long-lived luminescence materials and probes were developed (Connally and Piper, 2008; Bünzli, 2010; Yang et al., 2013; Zhang et al., 2018). Time-gated luminescence detection is known as one kind of time-resolved method, which can operate detecting gate after pulse excitation with a delay time (Connally and Piper, 2008; Lemmetyinen et al., 2014; Zhang et al., 2018). Therefore, the background signals caused by scattering and short-lived autofluorescence could be eliminated, and the signal-to-noise ratio would be improved significantly. Luminescence lifetime detection is another mainly used method, which can detect the luminescence decay rates by recording the luminescence intensity vs. pulse excitation (Cicchi and Pavone, 2011; Becker, 2012; Liu et al., 2019). Since the luminescence lifetimes of many probes are sensitive to microenvironments, lifetime imaging can exhibit significant differences that covered by luminescence intensity, and is more and more widely used in biological imaging to observe functional and molecular events (Connally and Piper, 2008; Berezin and Achilefu, 2010; Bünzli, 2010; Cicchi and Pavone, 2011; Strat et al., 2011; Becker, 2012; Damayanti et al., 2013; Yang et al., 2013; Grichine et al., 2014; Lemmetyinen et al., 2014; Lu et al., 2014; Bui et al., 2017; Luo et al., 2017; Wang et al., 2017; Zhang et al., 2018; Zhu et al., 2018b; Liu et al., 2019).

To achieve the time-resolved luminescence detection, the temporal resolution of related instruments should be high enough, since most luminescence probes exhibit luminescence lifetimes rang from sub-nanoseconds to milliseconds (Berezin and Achilefu, 2010; Bünzli, 2010; Yang et al., 2013; Zhang et al., 2018). With the development of pulse light sources, optical detectors and high-speed shutters, many instruments could achieve nanosecond-resolved luminescence detection (Krishnan et al., 2003; Urayama et al., 2003; Biskup et al., 2004; Connally et al., 2006; Qu et al., 2006; Sun et al., 2009; Gahlaut and Miller, 2010; Strat et al., 2011; Damayanti et al., 2013; Grichine et al., 2014; Hirvonen et al., 2014; Lu et al., 2014; Bergmann et al., 2016; Pominova et al., 2016; Bui et al., 2017; Luo et al., 2017; Wang et al., 2017; Zhu et al., 2018b; Liu et al., 2019). Photon sensitivity is another important factor in time-resolved luminescence detections. Since the luminescence decays exponentially after pulse excitation (Berezin and Achilefu, 2010; Bünzli, 2010; Yang et al., 2013; Zhang et al., 2018), the photon signals are significantly fewer than that under continuous-wave (CW) excitation. Although increasing the exciting power or exposure time could produce more luminescence photons, the photobleaching of many organic dyes would decrease the accuracy of the time-resolved luminescence measurements. Thus, it is necessary to increase the efficiency of photon detecting to improve the signal-to-noise ratio. Particularly in luminescence lifetime detections, a detecting cycle is usually divided into many intervals, each of which should have enough photons for luminescence lifetime analysis (Cicchi and Pavone, 2011; Becker, 2012; Liu et al., 2019).

Because of these requirements, high-speed and sensitive detectors, such as PMT (photomultiplier tube) (Strat et al., 2011; Grichine et al., 2014; Bui et al., 2017; Luo et al., 2017; Zhu et al., 2018b), SPAD (single-photon avalanche diode) (Damayanti et al., 2013; Lu et al., 2014; Wang et al., 2017), streak camera (Krishnan et al., 2003; Biskup et al., 2004; Qu et al., 2006; Bergmann et al., 2016; Pominova et al., 2016) or intensified camera (Urayama et al., 2003; Connally et al., 2006; Sun et al., 2009; Gahlaut and Miller, 2010; Hirvonen et al., 2014) are essential in many instruments for time-correlated single-photon counting (TCSPC). In addition, theses detectors always need to be synchronized with the ultrafast laser sources, leading to highly precise and complicated optical systems. Some reviews have summarized the principle and development of time-resolved luminescence detection techniques (Connally and Piper, 2008; Berezin and Achilefu, 2010; Bünzli, 2010; Cicchi and Pavone, 2011; Becker, 2012; Yang et al., 2013; Lemmetyinen et al., 2014; Zhang et al., 2018; Liu et al., 2019). Most of these techniques are mainly used in detecting nanosecond-delayed fluorescence. As the developments of phosphorescence, delayed fluorescence and upconversion luminescence materials, these materials could emit luminescence with a delay time over microseconds or milliseconds (Marriott et al., 1991, 1994; Verwoerd et al., 1994; Vereb et al., 1998; Connally et al., 2006; Connally and Piper, 2008; Bünzli, 2010; Gahlaut and Miller, 2010; Connally, 2011; Jin, 2011; Jin and Piper, 2011; Damayanti et al., 2013; Yang et al., 2013, 2019; Grichine et al., 2014; Hirvonen et al., 2014; Jin et al., 2014; Lu et al., 2014; Zhang et al., 2014, 2018; Bergmann et al., 2016; Pominova et al., 2016; Zheng et al., 2016; Zhu et al., 2016, 2018a,b; Bui et al., 2017; Chen T. et al., 2017; Wang et al., 2017; Sakiyama et al., 2018; Zhu and Shu, 2018, 2019; Deng et al., 2020; Liu et al., 2020), greatly reducing the requirement of temporal resolution and the cost of the instruments. Combining with the use of low-cost shutters and automatic synchronization methods, CW light sources and common cameras were successfully used to accomplish the time-resolved luminescence detection with the temporal resolution ranging from microseconds to milliseconds (Marriott et al., 1991, 1994; Verwoerd et al., 1994; Vereb et al., 1998; Connally, 2011; Jin, 2011; Jin and Piper, 2011; Jin et al., 2014; Zhang et al., 2014; Zheng et al., 2016; Sakiyama et al., 2018; Zhu and Shu, 2018, 2019; Zhu et al., 2018b; Yang et al., 2019; Deng et al., 2020). In this review, we will focus on these low-cost time-resolved techniques, especially chopper-based time-resolved luminescence detections.

## Overview of Time-Resolved Techniques

A time-resolved luminescence detection instrument is usually composed of a pulse source, an optical detector and a synchronous control component (Krishnan et al., 2003; Urayama et al., 2003; Biskup et al., 2004; Connally et al., 2006; Qu et al., 2006; Connally and Piper, 2008; Sun et al., 2009; Gahlaut and Miller, 2010; Cicchi and Pavone, 2011; Strat et al., 2011; Becker, 2012; Damayanti et al., 2013; Grichine et al., 2014; Hirvonen et al., 2014; Lemmetyinen et al., 2014; Lu et al., 2014; Bergmann et al., 2016; Pominova et al., 2016; Bui et al., 2017; Chen T. et al., 2017; Luo et al., 2017; Wang et al., 2017; Zhang et al., 2018; Zhu et al., 2018b; Liu et al., 2019, 2020). To achieve a temporal resolution of nanoseconds, many instruments equipped with a picosecond or even femtosecond laser (Krishnan et al., 2003; Urayama et al., 2003; Biskup et al., 2004; Qu et al., 2006; Sun et al., 2009; Grichine et al., 2014; Pominova et al., 2016; Bui et al., 2017; Luo et al., 2017; Zhu et al., 2018b), which is expensive. In order to increase the repetition rates and shorten the acquisition time, some lasers usually reach a frequency up to dozens of MHz (Damayanti et al., 2013; Luo et al., 2017), which is however not suitable for detecting luminescence with microseconds delay. Nowadays many TTL (transistor-transistor logic) modulated lasers and LEDs (light-emitting diode) could generate pulses within width range from nanoseconds to microseconds (Connally et al., 2006; Gahlaut and Miller, 2010; Hirvonen et al., 2014; Chen T. et al., 2017; Liu et al., 2020), which may produce more excited states per pulse than picosecond or femtosecond laser when exciting the long luminescence lifetime materials. The laser diodes and LEDs with various wavelength ranges are significantly cheaper than ultrafast lasers. And their emission stability and service life are better than that of mercury and xenon lamps.

Since many laser diode and LED sources are cheap and can be easily equipped in various optical systems, the optical detector becomes a key element in the instruments for time-resolved detection of long-lived luminescence. Various kinds of optical detectors used for time-resolved luminescence imaging are listed in Table 1. The temporal resolutions of PMT and SPAD can attain to nanoseconds, even to picoseconds with the sensitivity of single photon, so they are widely used to measure the luminescence lifetimes in TCSPC (Cicchi and Pavone, 2011; Strat et al., 2011; Becker, 2012; Damayanti et al., 2013; Grichine et al., 2014; Lu et al., 2014; Bui et al., 2017; Luo et al., 2017; Wang et al., 2017; Zhu et al., 2018b; Liu et al., 2019). But they could not distinguish photons in different space domain. To achieve luminescence lifetime imaging, these detectors and pulse lasers were usually equipped on the confocal laser scanning systems (Damayanti et al., 2013; Grichine et al., 2014; Lu et al., 2014; Bui et al., 2017; Wang et al., 2017), which have been popularized for luminescence microscopic imaging. However, the luminescence lifetime imaging based on point-by-point scanning is time consuming in detecting lifetimes over microseconds. The low repetition rate usually led to a long acquiring time over several minutes (Grichine et al., 2014; Lu et al., 2014; Bui et al., 2017). However, in some cases, samples with high concentration of luminescence particles can emit enough photons during one pulse cycle (Lu et al., 2014). In other cases, the scanning mode can be controlled flexibly to exclude the dark pixels for time-domain detection (Liu et al., 2019).

**Table 1:** Photo detectors used in time-resolved detections.

**Detector**	**Imaging method**	**Temporal resolution^a^**	**Luminescence lifetime detected**	**References**
PMT	Scanning	<30 ps	50 ps−1 ms	Strat et al., 2011; Grichine et al., 2014; Bui et al., 2017; Luo et al., 2017; Zhu et al., 2018b
SPAD	Scanning	<1 ns	0.8 ns–ms	Damayanti et al., 2013; Lu et al., 2014; Wang et al., 2017
Streak camera	Scanning	<1 ps	0.26 ns−1 ms	Krishnan et al., 2003; Biskup et al., 2004; Qu et al., 2006; Bergmann et al., 2016; Pominova et al., 2016
Intensified camera	Wide-field	0.2 ns	0.6 ns−1 ms	Urayama et al., 2003; Connally et al., 2006; Sun et al., 2009; Gahlaut and Miller, 2010; Hirvonen et al., 2014; Chen T. et al., 2017; Liu et al., 2020
Gated CMOS	Wide-field	<1 ns	0.45–4 ns	Ingelberts and Kuijk, 2015
Current-assisted CMOS	Wide-field	<1 ns	1.5–4 ns	Ingelberts and Kuijk, 2016
CMOS phase imager	Wide-field	0.11 ns	ns–μs	Guo and Sonkusale, 2012; Chen et al., 2015
SPAD imager CMOS	Wide-field	<1 ns	1–80 ns	Li et al., 2010; Ulku et al., 2019

a *The temporal resolution is represented by the gate time or the delay step of the detector*.

To improve the efficiency of time-resolved luminescence imaging, area-array detectors were developed to achieve wide-field time-resolved luminescence imaging of all the pixels (Urayama et al., 2003; Connally et al., 2006; Sun et al., 2009; Gahlaut and Miller, 2010; Li et al., 2010; Guo and Sonkusale, 2012; Hirvonen et al., 2014; Chen et al., 2015; Ingelberts and Kuijk, 2015, 2016; Chen T. et al., 2017; Ulku et al., 2019; Henderson et al., 2020; Liu et al., 2020). A common CCD or CMOS sensor is composed of an area-array of photosensitive silicon diodes, each of which could sense photons in microseconds (Henderson et al., 2020), but the actual frame rate is probably no more than hundreds of frames per second due to the limitation of readout time. To achieve both high gain and nanosecond resolution, micro channel plate (MCP) is developed and serves as a high-speed electronic shutter in the intensified cameras, which are widely used in wide-field time-gated imaging (Urayama et al., 2003; Connally et al., 2006; Sun et al., 2009; Gahlaut and Miller, 2010; Hirvonen et al., 2014; Chen T. et al., 2017; Liu et al., 2020). The streak tubes in streak cameras could distinguish photons in picoseconds (Krishnan et al., 2003; Biskup et al., 2004; Qu et al., 2006; Bergmann et al., 2016; Pominova et al., 2016). But these high-speed cameras are very expensive. In order to image the luminescence lifetime globally, some labs developed a variety of novel CMOS cameras (Henderson et al., 2020), which could implement time-gated control (Ingelberts and Kuijk, 2015, 2016), phase recording (Guo and Sonkusale, 2012; Chen et al., 2015; Ulku et al., 2019) or TCSPC (Li et al., 2010) on their sensor chips. Although these cameras could obtain luminescence lifetime images much faster than the scanning imaging, some of them were limited to detect mono or double exponential luminescence decay. Only a few of them have been used commercially, and their costs are still high.

In addition to the excitation sources and detectors, precise circuit systems with small timing jitter are usually used to synchronize the camera or shutter with the pulse excitation in many time-resolved detection systems (Krishnan et al., 2003; Urayama et al., 2003; Biskup et al., 2004; Connally et al., 2006; Qu et al., 2006; Sun et al., 2009; Gahlaut and Miller, 2010; Cicchi and Pavone, 2011; Strat et al., 2011; Becker, 2012; Hirvonen et al., 2014; Bergmann et al., 2016; Pominova et al., 2016; Chen T. et al., 2017; Luo et al., 2017; Liu et al., 2019, 2020). For detecting the delayed luminescence within nanoseconds, the related instruments are always composed of elements, such as ultrafast lasers, high-speed cameras and precise control circuits, which are very complicated and expensive to be popularized. However, there is no need to detect the microsecond-delay luminescence with nanosecond shutters, and a wide range of technologies were developed in the past decades to reduce the cost for time-resolved detection of phosphorescence and delayed fluorescence (Marriott et al., 1991, 1994; Verwoerd et al., 1994; Vereb et al., 1998; Connally, 2011; Jin, 2011; Jin and Piper, 2011; Jin et al., 2014; Zhang et al., 2014; Zheng et al., 2016; Sakiyama et al., 2018; Zhu and Shu, 2018, 2019; Zhu et al., 2018b; Yang et al., 2019; Deng et al., 2020).

**Figure 1:**
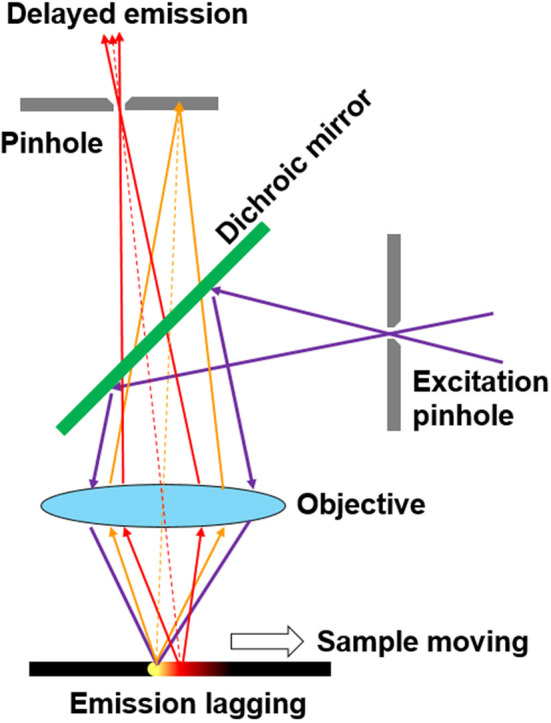
Schematic of pinhole shifting for scanning imaging of the delayed luminescence.

Some methods based on CW excitation were even developed to achieve time-resolved luminescence detection (Nuñez et al., 2013; Petrášek et al., 2016; Zhu, 2019). In 2008, Ramshesh et al. reported a luminescence lifetime imaging on a commercial confocal laser scanning microscope (Ramshesh and Lemasters, 2008). As long as the scanning speed is fast enough, the pixels would emit delayed luminescence lagging behind the excitation spot (Figure 1). By just shifting the pinhole of the microscopy, the phosphorescence lifetime imaging of a europium complex was accomplished with sub-millisecond resolution (Grichine et al., 2014). Although a pulsed Ti:Sapphire laser was used for multiphoton excitation, this method can be easily implemented on other confocal microscopes without additional attachments. However, the temporal resolution is limited by the scanning speed, which is in conflict with prolonging the exciting and dwelling time for producing enough photons. Additionally, the interferences caused by the scattering from the exciting spots further limited the improvement of signal-to-noise ratio. In 2016, Petrášek et al. reported a method which realized luminescence lifetime imaging in a standard confocal microscope without any modifications (Petrášek et al., 2016). During pixel scanning, the emitted luminescence would decrease as the scan velocity increased. Based on the dynamical processes of the excited states under CW excitation, the phosphorescence lifetime of a ruthenium complex was estimated to be about microseconds at each pixel.

For wide-field time-resolved imaging, low-cost shutters are used more and more widely for detecting luminescence with a delay over microseconds (Marriott et al., 1991, 1994; Verwoerd et al., 1994; Vereb et al., 1998; Connally, 2011; Jin, 2011; Jin and Piper, 2011; Jin et al., 2014; Zhang et al., 2014; Zheng et al., 2016; Sakiyama et al., 2018; Zhu and Shu, 2018, 2019; Zhu et al., 2018b; Yang et al., 2019; Deng et al., 2020). Notably, a mechanical chopper in combination with a highly sensitive camera is an alternative in time-gated luminescence imaging (Marriott et al., 1991, 1994; Vereb et al., 1998; Connally, 2011; Jin, 2011; Jin and Piper, 2011; Jin et al., 2014; Zhang et al., 2014; Zheng et al., 2016). The details of the time-resolved luminescence imaging based on some mechanical choppers are shown in Table 2. Compared with electro-optic or acousto-optic choppers, the mechanical choppers could provide 100% modulation, which is independent of wavelength (Selzer and Yen, 1976). Although the switching time of commercial mechanical choppers is always shorter than electronic switches and intensifiers in many high-speed detectors, they could be well used in time-resolved luminescence imaging and spectrum detection with a delay time over microseconds (Marriott et al., 1991, 1994; Vereb et al., 1998; Connally, 2011; Jin, 2011; Jin and Piper, 2011; Jin et al., 2014; Zhang et al., 2014; Zheng et al., 2016; Sakiyama et al., 2018; Zhu and Shu, 2018, 2019; Zhu et al., 2018b; Yang et al., 2019; Deng et al., 2020). Now, we will review some principles and applications of mechanical choppers used in time-resolved luminescence detection, particularly the auto-phase-locked method developed in our previous work. Finally, we will give a brief outlook for the auto-phase-locked time-resolved luminescence detection systems, and hope some improvements of the systems and original applications can be realized in future.

**Table 2:** Chopper-based time-resolved luminescence imaging.

**Light source**	**Camera**	**Synch control**	**Temporal resolution^a^**	**Luminescence lifetime detected**	**References**
488 nm laser	CCD	Circuit	50 μs	~ 1 ms	Marriott et al., 1991
Mercury lamp	CCD	Circuit	210 μs	0.56–0.89 ms	Marriott et al., 1994
UV LED	CCD	Circuit	11–16 μs	>0.1 ms^b^	Jin, 2011; Jin and Piper, 2011
Xenon lamp	Color CCD	Circuit	≤ 88 μs	>0.1 ms^b^	Zhang et al., 2014
980 nm laser	EMCCD	Circuit	≤ 23 μs	μs–ms^b^	Zheng et al., 2016
UV LED	CCD	Auto	<260 μs	0.2–0.7 ms	Connally, 2011
980 nm laser	CCD	Auto	50–200 μs	< ms^b^	Zhu et al., 2018b
405 nm laser	Color CCD	Auto	≤ 10 μs	2–60 μs	Zhu and Shu, 2019
405 nm laser	CCD	Auto	≤ 128 μs	~200 μs	Sakiyama et al., 2018
405 nm laser	sCMOS	Auto	≤ 20 μs	16 μs	Yang et al., 2019

a *The temporal resolution is represented by the switching time or the delay time*.

b *These lifetimes were estimated from the properties of similar luminescence materials*.

## Introduction of Mechanical Choppers

**Figure 2:**
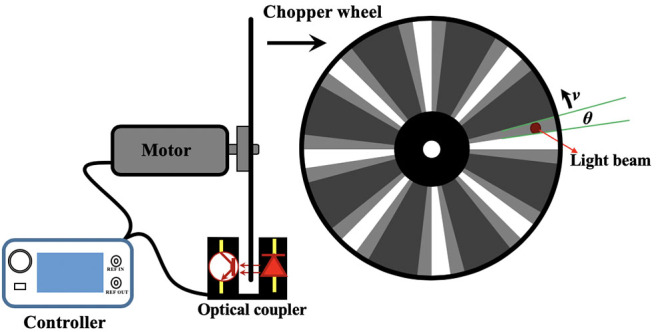
Diagram of commercial chopper and chopper wheel.

A mechanical chopper is usually composed of a motor which could drive a rotating wheel, mirror or prism to modulate a continuous light beam into light pulses (Hoffmann and Jovin, 1971; Selzer and Yen, 1976; Gembicky et al., 2005; Förster et al., 2015). As shown in Figure 2, the rise or fall time of a pulse chopped by a wheel with slots could represent the temporal resolution, which can be expressed as:

$$\begin{array}{r} {res}_{time}=\frac{r_{beam}}{v} \end{array}$$

where *r*_*beam*_ and *v* represent the radius of the light beam and the orbital velocity of the disk, respectively. In order to accomplish a temporal resolution as high as possible, a faster rotating speed and bigger wheel is preferred. However, the rotating speed is limited by centrifugal stress and the torque required to overcome aerodynamic drag (Wenthen and Snowman, 1973). Therefore, the wheel size is limited by its tensile strength to prevent it from bursting apart.

In 1971, Hoffmann and Jovin constructed a chopper which can rotate a prism up to 7,000 rounds per second (RPS), further modulating a continuous laser beam to produce nearly 0.5-ns rise-time rectangular-shaped pulses of laser light (Hoffmann and Jovin, 1971). Then in 1976, Selzer and Yen used an air turbine handpiece to develop a chopper which can rotate as fast as 8,300 RPS and span the region from 500 Hz to 300 kHz with a single blade change (Selzer and Yen, 1976). Although the chopper rotated fast, the wheel diameter is only 12.5 mm, so as to reduce the torque load on the turbine. For wheels with diameter over 100 mm, many lab made choppers could spin over hundreds Hz. A 339-mm-diameter wheel with thickness from 30 mm at the center to 0.5 mm at outermost rim could rotate at 998 RPS, which was used to extract picosecond X-ray pulses (Förster et al., 2015).

Although many lab made choppers could rotated over 1,000 RPS (Hoffmann and Jovin, 1971; Selzer and Yen, 1976; Gembicky et al., 2005), the commercial choppers are usually designed to have a max rotating speed of only 100 RPS. Some commercial choppers can rotate at 270 RPS and its frequency can be up to 120 kHz with 445-slot blade (Model-310CD, Scitec Instruments Ltd.). Therefore, the temporal resolutions of many chopper-based time-resolved detection systems were limited to microseconds. To maintain the chopping speed and phase-locking, many choppers have integrated some electrical circuitries to monitor and control the wheel rotation. The commercial choppers usually have a controller which can get the reference signal by the optical switch at the edge of the wheel (Figure 2), and convert optical signals into TTL signals, further accomplishing the synchronization with other equipment, such as light sources and signal generators for many optical measurements.

## The Chopper-Based Time-Resolved Detection by Electrical Synchronization

**Figure 3:**
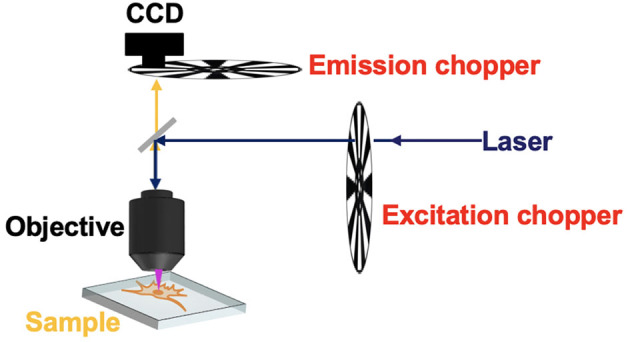
Schematic of the time-gated phosphorescence microscope equipped with two choppers.

In many time-resolved luminescence detection systems, the detection shutter and excitation pulse should be synchronized. For the CW light sources, one chopper could be used to generate pulsed excitation, while another chopper acted as a shutter synchronized to the first one for time-gated luminescence detection (Figure 3) (Marriott et al., 1991, 1994; Vereb et al., 1998). By using different exciting sources, Marriott et al. used this method to image the delayed luminescence of acridine orange (Marriott et al., 1991) and a Europium (III) complex (Marriott et al., 1994), respectively. The phase difference between the two choppers could be adjusted by the controllers to acquire a series of images with different delay time, so the luminescence lifetime can be measured at each pixel.

Nowadays, many laser and LED sources can be modulated by TTL signals, hence the reference signals of the choppers can be used to achieve the synchronization of excitations to the chopping cycles. In 2011, Jin used the clock signal from the chopper to achieve the synchronization of the gated detection cycle in a time-gated luminescence microscopy, which was used to achieve real-time direct-visual inspection and true color imaging of *Cryptosporidium parvum* labeled by europium and terbium complexes (Jin, 2011; Jin and Piper, 2011). However, the power or the frequency of currently available UV LEDs at 300–340 nm was not high enough for exciting some terbium complexes. They used a high-power xenon flash lamp synchronized to accomplish dual-color visualization of the time-gated phosphorescence (Zhang et al., 2014). Then this method was applied to synchronize a 980 nm laser to accomplish time-gated upconversion luminescence imaging of mice by Zheng et al. (2016). The lanthanide upconversion nanoparticles always require excitation by high-power lasers over W/cm^2^, which usually cause substantial scattering even with the used of filters under CW excitation. While in time-gated imaging, high-contrast upconversion luminescence was visualized with a high signal-to-noise ratio.

## The Chopper-Based Time-Resolved Detection by Auto Synchronization

The auto synchronization method was developed to avoid phase mismatch between excitation and detection. This method was easier to implement than electrical synchronization, since the excitation and detection paths were modulated by one chopper wheel simultaneously. The phase difference of excitation and detection could be locked even though there were some frequency jitters, which made the method called auto-phase-locked time-resolved detection (Connally, 2011; Sakiyama et al., 2018; Zhu and Shu, 2018, 2019; Zhu et al., 2018b; Yang et al., 2019; Deng et al., 2020). Even before the spread of motor technology, a manual chopper consists of two coaxial wheels was invented by Becquere in 1859 for the detection of phosphorescence with millisecond-delay (Berezin and Achilefu, 2010). The two wheels with holes were not lined up, so the sample between the two wheels was excited by a beam of incident sunlight through one hole, and the phosphorescence was viewed through the other hole. The coaxial wheels ensured the phase difference and achieved a temporal resolution of 0.8 ms.

**Figure 4:**
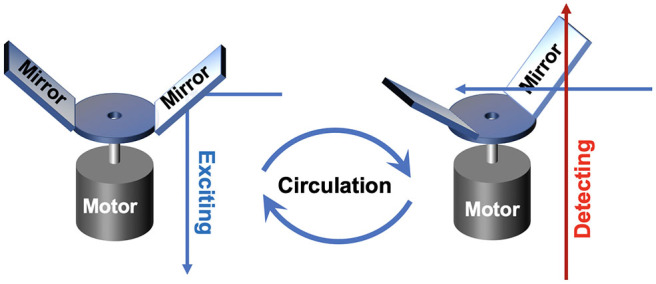
Schematics of gated autosynchronous luminescence detection with the special designed chopper in excitation and detection process.

In 2011, an auto synchronous luminescence time-resolved microscopy equipped with a special chopper was designed by Connally (2011), as shown in Figure 4. Two mirror finishes were fabricated by highly polishing the aluminum rotor face and lied at an angle of 45 to the motor axis with a radial sweep at the perimeter of 90, further reflecting the excitation light beam periodically. Then the delayed luminescence emitted from the sample could pass the chopper at intervals between reflections, which could precisely synchronize the excitation pulse, the resolving period, and the detection phase. A compact high-power UV LED was employed as the excitation source, and the images of Giardia lamblia cysts indirectly labeled with a europium chelate/streptavidin conjugate were captured successfully.

**Figure 5:**
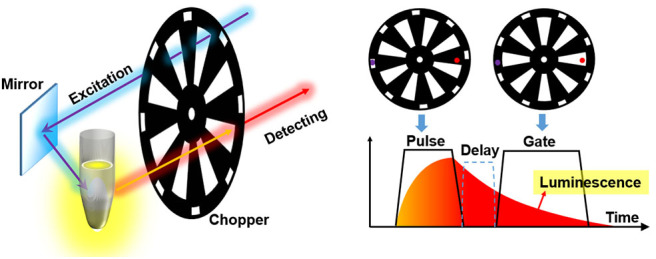
Schematics of gated autosynchronous luminescence detection by a chopper wheel. The diagram (right) shows the time-gated luminescence detection when the wheel rotating.

To accomplish the auto synchronization with one chopper wheel, Pollak and Maszkiewicz claimed a method in a patent in 1990 (Pollak and Maszkiewicz, 1990). The excitation path was fixed nearly parallel to the detecting path, so that two paths could pass through the slots with different rotation radiuses of only one wheel at the same time, where the outer slots were used to generate pulse excitation and the inner slots act as a detecting shutter (Figure 5). The phases of the excitation and detection can be synchronized automatically as long as their optical paths are fixed without the requirement of complex phase matching circuitry or control system. The delayed luminescence could pass the chopper wheel when the excitation was blocked with appropriate phase difference even if there were some frequency jitters.

The method was also used in a time-gated luminescence spectrometer (Saito et al., 2005). Similarly, the wheels with single circle of slots can also be used to synchronize the excitation and detecting, and the gate time is nearly the same as the pulse width (Sakiyama et al., 2018; Zhu and Shu, 2018, 2019; Zhu et al., 2018b; Yang et al., 2019; Deng et al., 2020). Since the auto synchronization greatly simplified the instruments and reduced the cost, this auto-phase-locked time-resolved technique has been well used in time-gated luminescence imaging (Sakiyama et al., 2018; Zhu et al., 2018b; Yang et al., 2019), spectrometer (Zhu and Shu, 2018) and luminescence lifetime detection (Zhu and Shu, 2019; Deng et al., 2020).

## Time-Gated Luminescence Detection and Imaging

The low-cost and auto-phase-locked time-gated luminescence measurement system can be used for time-gated luminescence spectra measurement, which can decrease the scattering interference from exciting light effectively (Sakiyama et al., 2018; Zhu et al., 2018b; Yang et al., 2019). With different types of excitation light sources used, both of background-free downconversion and upconversion delayed luminescence spectra detection can be achieved successfully.

**Figure 6:**
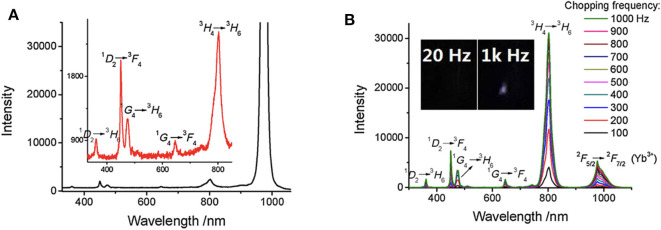
Steady-state emission spectra **(A)** and time-gated emission spectra **(B)** of Tm/Yb doped nanocrystals (Tm 2%, Yb 18%). λ_ex_ = 980 nm. The exciting power is about 1 W. Inset: the delayed luminescence emission at different chopping frequency. Reprinted from Zhu et al. (2018b). Copyright (2018), with permission from Elsevier.

As shown in Figure 6, the spectra of Tm/Yb doped NaYF_4_ nanocrystals at different chopping frequency were measured (Zhu et al., 2018b). Compared to the steady-state spectra, most scattering signals of the exciting laser at about 980 nm were eliminated effectively at time-gated mode (Figure 6A). With a low background, there was a clear emission peak caused by the radiative transition of Yb^3+^ (^2^F_5/2_→^2^F_5/2_), while it was covered by the scattering light in the steady state spectrum. No obvious upconversion luminescence was detected at a low chopping frequency, and the delayed luminescence enhanced significantly as the chopping frequency increased (Figure 6B), which is resulted from that the delay time was shorten as the chopping frequency increased. This method was also used to measure the downconversion time-gated luminescence spectra of some fluorescence molecules with a delay time of only a few microseconds (Zhu and Shu, 2018).

**Figure 7:**
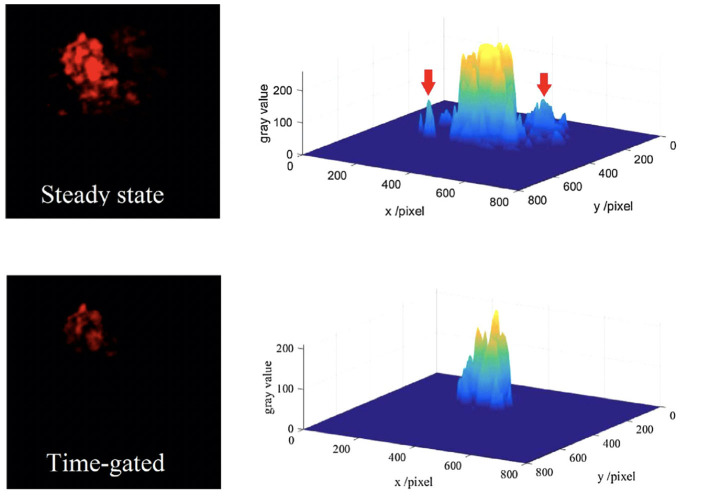
The red channels and their gray distributions of the microscopic images at steady state (the inset arrow indicating some scattering signals) and time-gated with a chopping speed of 1,000 Hz. Reprinted from Zhu et al. (2018b). Copyright (2018), with permission from Elsevier.

The auto-phase-locked method was then used to construct an upconversion luminescence microscopy for time-gated luminescence imaging. The delayed luminescence of the Murine B16 melanoma cells incubated by water-dispersed Er/Yb doped NaYF_4_ nanocrystals was captured by a color CCD (Zhu et al., 2018b). The background of the steady-states was much higher than that of the time-gated images, though the images in steady state mode showed brighter luminescence (Figure 7). There were even some signals outside of the cell which was probably the scattering signals from the exciting light, while the time-gated image exhibited a clean background despite the high exciting power. The auto-phase-locked method was also used to accomplish the time-gated imaging of silicon quantum dots with downconversion long-lived luminescence in biological tissue, and greatly increased the signal-to-noise ratio (Sakiyama et al., 2018; Yang et al., 2019).

## Luminescence Lifetime Detection and Imaging

**Figure 8:**
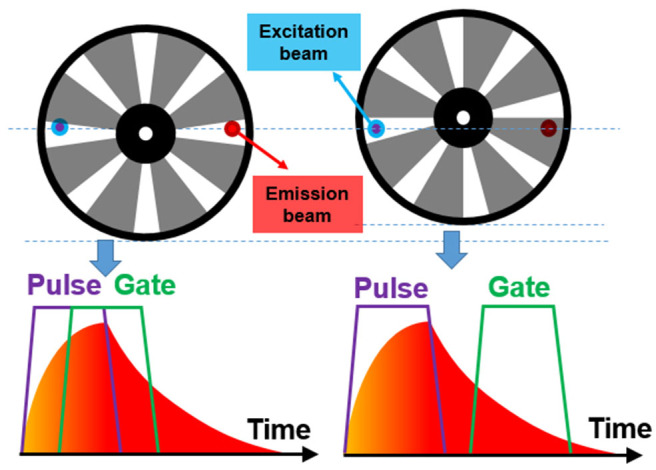
Schematics of the phase modulation by changing the altitude of the chopper wheel.

**Figure 9:**
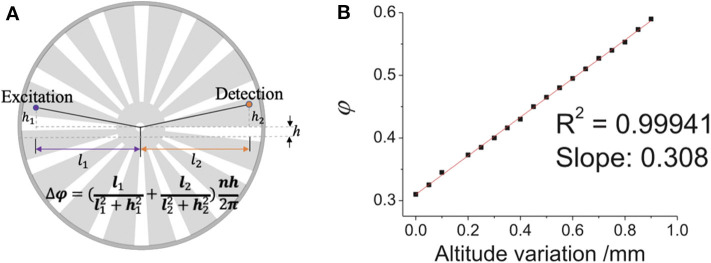
Diagram of the change of the chopper altitude. **(A)** Schematic diagram of adjusting the phase difference between pulse excitation (left spot) and shutter (right spot). **(B)** The phase difference (ϕ) vs. the altitude variation of the chopper wheel (the delay time Δ*t* = *T*∙φ). Reprinted from Zhu and Shu (2019). Copyright (2019), with permission from Elsevier.

Although time-gated method can detect delayed luminescence, it can hardly distinguish the delayed signals with similar luminescence lifetimes, to measure which the luminescence intensity change in time domain should be recorded. Therefore, we developed a simple way to adjust the delay time to achieve luminescence lifetime analysis on the time-gated system. Since the chopper can be placed on a displacement platform, it can be moved vertically, hence the phase difference between pulse excitation and shutter can be adjusted easily and flexibly (Figure 8). As shown in Figure 9A, when the altitude of the chopper changed with a variation of *h* which is much less than the distance between the light path and the chopper center (*h*≪*l*_1_, *l*_2_), the variation of the phase difference (φ) can be expressed as (Zhu and Shu, 2019):

$$\begin{array}{r} \Delta \varphi =\frac{\Delta \theta }{2\pi /n}=\left(\frac{l_{1}}{l_{1}^{2}+h_{1}^{2}}+\frac{l_{2}}{l_{2}^{2}+h_{2}^{2}}\right)\frac{nh}{2\pi } \end{array}$$

where *n* represents the number of the chopper slots and Δθ is central angle variation. By using an oscilloscope equipped with two parallel silicon photodiodes to measure the phase difference (Figure 9B), the phase difference variation (Δφ) was proved to be linear with the altitude variation of the chopper (*h*), which also means that the delay time (Δ*t*) was also linear with the altitude variation of the chopper, since the delay time can be calculated by:

$$\begin{array}{r} \Delta t=T∙\Delta \varphi \end{array}$$

where T is the chopping period. Herein, a simplified method was developed later for measuring the delay time with no additional detectors. Firstly, the altitude variation (H) of one period which corresponds to 2π phase difference was measured. Therefore, the change of the delay time can also be calculated by (Deng et al., 2020):

$$\begin{array}{r} \Delta t=T∙\frac{h}{H} \end{array}$$

**Figure 10:**
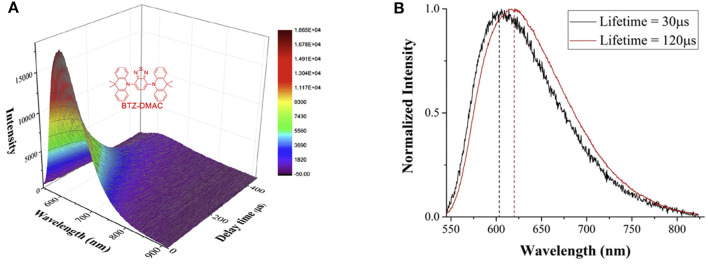
**(A)** Time-gated luminescence spectra of BTZ-DMAC measured at different delay time at chopping frequency of 1 kHz. Inset: the structure of BTZ-DMAC. **(B)** The luminescence spectra of the two excited states with different lifetimes. Reprinted from Deng et al. (2020). Copyright (2020), with permission from Elsevier.

Combining the phase difference adjustment with the time-gated detection, the spectrally resolved luminescence lifetimes can be measured. As shown in Figure 10A, the spectra of a classical TADF (thermally-activated delayed fluorescence) molecule, BTZ-DMAC, at different delay time were recorded by a spectrograph (Deng et al., 2020). Two long-lived excited states with lifetimes of 30 and 120 μs, respectively, were revealed by fitting the integral of luminescence signals at all wavelengths at different delay time. It is easy to find that the longer-lifetime excited state occupies a larger proportion, and the maximum intensity of the two excited states were gained at 608 and 616 nm, respectively (Figure 10B), suggesting a small energy gap between different excited states probably caused by conformation differences.

**Figure 11:**
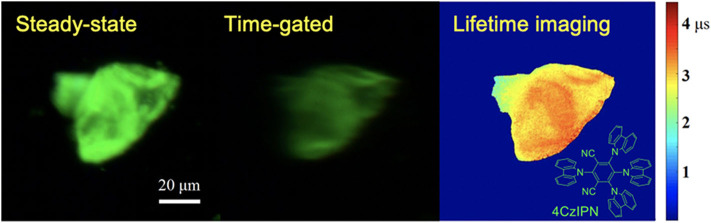
Steady-state, time-gated and luminescence lifetime imaging of 4CzIPN. Inset: the structure of 4CzIPN. Reprinted from Zhu and Shu (2019). Copyright (2019), with permission from Elsevier.

Using a CCD camera as a detector, global luminescence lifetime imaging was achieved with a temporal resolution of microseconds (Zhu and Shu, 2019). As the phase difference was adjusted by changing the altitude of the chopper, a series of time-gated luminescence images with different phase difference can be gained and used for luminescence lifetime imaging of a typical thermally activated delayed fluorescence materials, 4CzIPN. As shown in Figure 11, the time-gated image exhibited low scattering light and background which was accomplished by the auto-phase-locked principle. The delay time increased as the phase difference increased, further leading to a decrease of the luminescence intensity. The luminescence lifetimes at each pixel were estimated by fitting the luminescence intensity represented by the grayscales at each pixel at different delay time with the exponential function. In this way, the luminescence lifetime imaging of 4CzIPN powders could be constructed, as shown in Figure 11, where different colors were used to represent different lifetimes ranging from 2 to 3.5 μs. Practically, the delay time was adjusted in steps much smaller than the gate width (>50 μs) in these experiments, resulting in overlapping gates. However, the lifetimes within a few microseconds could be well-measured, because a moving resolution of 10 μm of the stage could achieve a temporal resolution of sub-microseconds for the delay control. In addition, the rise and fall time of the shutter was proved to have little effect on the exponential fitting at a constant chopping frequency (Zhu and Shu, 2019). Compared to luminescence lifetime imaging systems based on confocal laser scanning systems (Cicchi and Pavone, 2011; Strat et al., 2011; Becker, 2012; Damayanti et al., 2013; Grichine et al., 2014; Lu et al., 2014; Bui et al., 2017; Luo et al., 2017; Wang et al., 2017; Zhu et al., 2018b; Liu et al., 2019), this method will save much time in luminescence lifetime imaging of long-lifetime luminescence.

## Challenges and Prospects

The auto-phase-locked method exhibited great potential in time-resolved luminescence detection and imaging. Since the method could significantly reduce the phase jitters, the temporal resolution is mainly limited by the rotational speed of the chopper. To our knowledge, the fastest orbital velocity of a wheel driven by a chopper was 1,063 m/s (Förster et al., 2015), suggesting a rise time of 95 ns for opening a 0.1-mm-wide light beam, which can be used to test most phosphorescent and delayed luminescence (Bünzli, 2010; Yang et al., 2013; Zhang et al., 2018). Another important feature of this method is that the temporal accuracy of the delay time or the phase difference depends on the moving accuracy of the stages. This method is absolutely different with the phase modulation based on circuit control. With a high-precision stage, the time step could be much shorter than the chopper shutter. Combining with the ultrafast mechanical chopper, the auto-phase-locked method may be used to measure the luminescence lifetimes of tens of nanoseconds.

Despite some defects of the mechanical chopper, such as the rotating instability, there are no other shutters with both low cost and high modulation depth for broadband light. Some MEMS (micro electromechanical system) choppers may reach a much higher frequency than mechanical choppers (Chao et al., 2007; Tsuchiya et al., 2016; Chen P. et al., 2017; Chen T. et al., 2017), but they were mainly used for modulating light sources. These vibration-based choppers are safer to be used than the ultrafast mechanical chopper. If two incoherent light beams were modulated by a MEMS chopper, the auto synchronization could be implemented for time-gated luminescence detection.

As the developments and applications of luminescence materials, such as phosphorescence, delayed fluorescence and upconversion luminescence materials become more and more widely, we believe the time-resolved techniques would be more and more used for measuring luminescence lifetimes and spectra. And the time-resolved luminescence imaging would be a powerful method for their applications in biological imaging and detections. To detect the long-lived luminescence of these materials, there is no need to use high-speed detectors and ultrafast light sources. A laser or LED modulated by a chopper with a pulse width of a few microseconds may be more suitable than ultrafast lasers to excite the luminescence with lifetime over sub-millisecond. Compared to the approaches based on electrical synchronization, the auto synchronization method avoids the phase jitters caused by the mechanical jitters of the choppers. Because the auto-phase-locked method needs no synchronization circuits, the control element could be simple, which could be propitious to construct miniaturized apparatuses for time-resolved detection.

In addition to the development of device hardware, the backward analysis of the dynamical photophysical process may be used to develop novel methods for time-resolved luminescence detection. It is possible to use Petrášek's method (Petrášek et al., 2016) on some chopper-based systems for luminescence lifetime imaging by setting no phase difference between excitation and detection. By changing the chopping frequency instead of the phase difference, the approximations of the luminescence lifetimes may be estimated. The ideas and principles are still being developed. We believe the methods as well as choppers would be useful to construct low-cost instruments for microsecond-resolved luminescence detection and imaging.

## Author Contributions

QD wrote the manuscript and prepared the figures. ZZ applied for the permission of the use of the images in other published journals and helped to prepare the figures. XS took the primary responsibility for communication with the journal and editorial office during the submission process, throughout peer review and during publication. All authors contributed to the article and approved the submitted version.

## Conflict of Interest

The authors declare that the research was conducted in the absence of any commercial or financial relationships that could be construed as a potential conflict of interest.
